# Microarray analysis revealing common and distinct functions of promyelocytic leukemia protein (PML) and tumor necrosis factor alpha (TNF*α*) signaling in endothelial cells

**DOI:** 10.1186/1471-2164-13-453

**Published:** 2012-09-04

**Authors:** Xiwen Cheng, Hung-Ying Kao

**Affiliations:** 1 Department of Biochemistry, School of Medicine, Case Western Reserve University (CWRU) and the Comprehensive Cancer Center of CWRU and University Hospital of Cleveland (UHC), Cleveland, OH 44106, USA

**Keywords:** PML, TNF*α*, Endothelial cells, Microarray, Inflammation, Cell adhesion

## Abstract

**Background:**

Promyelocytic leukemia protein (PML) is a tumor suppressor that is highly expressed in endothelial cells nonetheless its role in endothelial cell biology remains elusive. Tumor necrosis factor alpha (TNF*α*) is an important cytokine associated with many inflammation-related diseases. We have previously demonstrated that TNF*α *induces PML protein accumulation. We hypothesized that PML may play a role in TNF*α *signaling pathway. To identify potential PML target genes and investigate the putative crosstalk between PML’s function and TNF*α *signaling in endothelial cells, we carried out a microarray analysis in human primary umbilical endothelial cells (HUVECs).

**Results:**

We found that PML and TNF*α *regulate common and distinct genes involved in a similar spectrum of biological processes, pathways and human diseases. More importantly, we found that PML is required for fine-tuning of TNF*α*-mediated immune and inflammatory responses. Furthermore, our data suggest that PML and TNF*α *synergistically regulate cell adhesion by engaging multiple molecular mechanisms. Our biological functional assays exemplified that adhesion of U937 human leukocytes to HUVECs is co-regulated by PML and TNF*α *signaling.

**Conclusions:**

Together, our study identified PML as an essential regulator of TNF*α *signaling by revealing the crosstalk between PML knockdown-mediated effects and TNF*α*-elicited signaling, thereby providing novel insights into TNF*α *signaling in endothelial cells.

## Background

Promyelocytic leukemia protein (PML) is a tumor suppressor protein, originally identified as a fusion partner of the retinoid acid receptor (RAR) gene characteristic of chromosomal translocation involved in acute promyelocytic leukemia (APL). PML is enriched in distinct nuclear sub-domains known as PML nuclear bodies (NBs) [1]. Hitherto, more than 160 proteins have been reported to constitutively or transiently reside in PML NBs. Recent studies suggest that PML is involved in the regulation of various cellular processes including transcription, cell cycle, post-translational modification, anti-viral responses, DNA damage repair, apoptosis, and cell adhesion in response to extracellular stimuli [2-8]. In addition, studies showed that PML protein accumulation is down-regulated in many cancer types suggesting that PML is a tumor suppressor [9]. A tissue profiling study showed that PML is highly expressed in endothelial cells (ECs) and tissues with inflammation [1], but its physiological significance in these contexts remains elusive.

The tumor necrosis factor alpha (TNF*α*), secreted by activated immune cells including macrophages during inflammation, elicits a cascade of cellular signaling events in ECs [10-12], such as up-regulation of leukocyte adhesion molecules and increased endothelial permeability. TNF*α *was initially described as being capable of shrinking tumor size, however later studies suggested that it may stimulate tumor growth by promoting inflammation [13]. Thus, pharmaceutical application of TNF*α* or TNF*α* inhibitors in cancer treatment remains debatable [12]. To this end, we have recently investigated the role of PML on angiogenesis in TNF*α*-stimulated ECs and showed that TNF*α* induces PML expression and PML is important for TNF*α*-suppressed angiogenesis [14,15]. These observations suggest that there may be complex relationship between PML and TNF*α *signaling.

To further dissect the role of PML in ECs and especially in the presence of TNF*α *signaling, we took an unbiased systematic approach. We knocked down PML by two independent siRNAs with a non-targeting siRNA as control in the presence or absence of a mild dose of TNF*α* in primary human umbilical vein endothelial cells (HUVEC) and carried out gene expression microarray studies. Following identification of the significantly altered genes, we performed extensive functional analysis and identified an intricate pattern of crosstalk between PML and TNF*α *signaling.

## Results

### Identification of PML target genes, TNF*α *responsive genes and synergistically regulated genes by PML and TNF*α*

**Figure 1:**
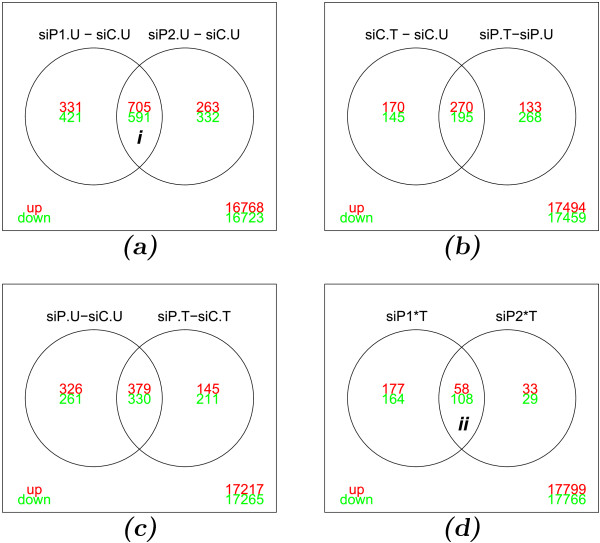
**Venn diagrams of significantly affected genes.** (**a**) Significantly affected genes following PML knockdown by two independent siRNAs (siP1, siP2) compared to control siRNA (siC) in HUVECs without TNF*α *treatment. The intersection of both circles (*i*) is considered significantly affected genes by PML knockdown (designated as “siP.U-siC.U”). (**b**) Comparison between TNF*α *responsive genes (“siC.T-siC.U”) and TNF*α *responsive genes when PML was knocked down (“siP.T-siP.U”). (**c**) Comparison between PML-knockdown responsive genes in the absence of TNF*α *treatment (*i*, “siP.U-siC.U” from **a**) and in the presence of TNF*α *treatment (“siP.T-siC.T”). (**d**) Identification of genes interactively regulated by PML knockdown (two siRNAs, siP1 and siP2) and TNF*α *treatment. The intersection of both circles (*ii*) is considered significantly affected interaction genes. Untreated samples, “U”; TNF*α *treated samples, “T”; comparison between two samples, “-”; interaction effects, “∗”. Numbers in the circles, the number of significantly altered genes (>1.5*fold*,*q *< 0.05) by the indicated comparison. Numbers in the outer box, the number of unchanged genes; up-regulation, “up” shown in red; down-regulation, “down” shown in green.

To identify potential PML target genes, we transiently transfected PML targeting siRNAs into HUVECs to knock down PML expression prior to the microarray gene expression analyses. Because siRNAs can have off-target effects, we used two independent PML siRNAs (“siP1” and “siP2”) targeting different regions of PML transcripts with duplicate samples to help eliminate false target genes. We considered a gene to be significantly altered in responsive to PML knockdown if was identified by both siRNAs. Thus, using >1.5 fold and a *q *< 0.05 as cut-off parameters, we identified 705 genes up-regulated (UP) and 591 genes down-regulated (DOWN) as a result of PML knockdown in the absence of TNF*α *treatment in HUVECs (Figure 1a, the intersection of both circles, *i*, designated as “siP.U-siC.U” hereinafter). Using similar parameters, we also identified genes whose expression is significantly altered following 20 h of TNF*α *treatment in wild type cells (Figure 1b, “siC.T-siC.U”, left circle). Our data show that totally 440 genes were up-regulated, whereas 340 genes were down-regulated in response to TNF*α* treatment (Figure 1b, left circle). Because TNF*α *potently induces PML expression [14,15], we also carried out microarray studies in which PML was knocked down with or without TNF*α* treatment. When PML was knocked down, TNF*α *induced 403 genes and repressed 463 genes (Figure 1b, “siP.T-siP.U”, right circle). Among these genes, 270 (UP) and 195 (DOWN) are common TNF*α *responsive genes, regardless of whether PML was present or not (Figure 1b, the intersection of both circles). We identified 145 (UP) and 211 (DOWN) genes that were responsive to PML knockdown only in the presence of TNF*α *(Figure 1c, the relative complement of “siP.U-siC.U” in “siP.T-siC.T”). Because of the factorial design of our experiments, we were able to estimate the interaction effects of PML knockdown and TNF*α* treatment in HUVECs that is designated as siP∗*TNFα *and calculated as *siP*∗*T *= (*siP.T*−*siC.T*)−(*siP.U*−*siC.U*) or equally *siP*∗*T *= (*siP.T*−*siP.U*)−(*siC.T*−*siC.U*). Similarly as in Figure 1a, we identified 166 such genes by two different siRNAs (“siP1” and “siP2”), of which 58 genes positively responded while 108 genes showed negative interaction effect following PML knockdown and TNF*α *treatment (Figure 1d, the intersection of both circles, *ii*). Full gene lists are shown in Additional file 1.

### Gene Ontology analysis of PML target genes and TNF*α *responsive genes

**Figure 2:**
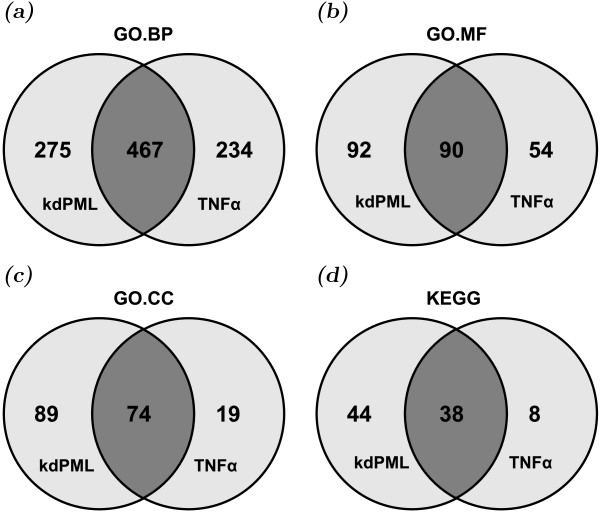
**Functional ontology analyses of PML knockdown responsive genes and TNF*α *treatment affected genes.** The identified gene lists were analyzed by the Hyper-Geometric test (*q *< 0.01) as described in Methods to identify the over-represented terms of (**a**) GO.BP, (**b**) GO.MF, (**c**) GO.CC and (**d**) KEGG pathways affected by PML knockdown (“kdPML”) and TNF*α *treatment (“TNF*α*”). Two parental GO terms were removed to reduce redundancy of the GO term definition. GO.BP, Gene Ontology Biological Process; GO.MF, Gene Ontology Molecular Function; GO.CC, Gene Ontology Cellular Component; KEGG, Kyoto Encyclopedia of Genes and Genomes.

Using the genes described in Figure 1, we carried out functional ontology analyses and identified the biological functions significantly affected (*q*<0.01) by PML knockdown and TNF*α *treatment through hypergeometric tests using the Gene Ontology (GO) (Figure 2a–c). We found that PML knockdown and TNF*α *treatment affected a considerable number of common biological functions. The top 25 Gene Ontology Biological Processes (GO.BP) commonly affected by both treatments are listed in Table 1. Specifically both treatments affected various *metabolic* processes, *cell communication*, *signal transduction*, multiple *biosynthetic processes*, *gene expression*, *transport*, and *multicellular organismal development*. Full lists of significantly affected gene ontology information are found in Additional file 2.

**Table 1:** **Top 25 GO.BP affected by both PML knockdown and TNF*α *treatment**

**Gene Ontology Biological Process (GO.BP)**	**PML Knockdown**		**TNF*α***	
	**Gene number**	***q *value**	**Gene number**	***q *value**
Cellular metabolic process	539	3.04E-116	296	9.50E-52
Primary metabolic process	527	4.97E-104	287	1.06E-44
Regulation of cellular process	449	1.71E-75	301	1.92E-60
Macromolecule metabolic process	441	1.25E-81	237	7.06E-34
Biopolymer metabolic process	433	7.96E-80	230	6.54E-32
Cellular macromolecule metabolic process	413	1.71E-78	219	1.87E-31
Cellular biopolymer metabolic process	403	4.47E-75	210	2.56E-28
Biosynthetic process	294	9.75E-49	160	3.39E-21
Cell communication	288	5.37E-52	219	2.97E-56
Cellular biosynthetic process	288	9.70E-48	155	4.47E-20
Nitrogen compound metabolic process	274	6.66E-39	144	7.73E-15
Signal transduction	260	4.09E-45	201	3.61E-51
Protein metabolic process	253	7.52E-57	141	2.06E-27
Nucleobase, nucleoside, nucleotide and nucleic acid metabolic process	240	9.74E-31	119	1.98E-09
Macromolecule biosynthetic process	230	5.36E-35	120	2.81E-13
Cellular protein metabolic process	229	1.32E-56	125	4.20E-26
Cellular macromolecule biosynthetic process	228	1.25E-34	117	2.02E-12
Gene expression	211	5.35E-24	118	3.60E-11
Cellular biopolymer biosynthetic process	209	3.83E-30	106	1.96E-10
Biopolymer biosynthetic process	209	5.59E-30	106	2.34E-10
Transport	201	1.57E-37	124	1.35E-23
Regulation of metabolic process	197	1.26E-21	137	3.76E-20
Multicellular organismal development	193	5.30E-36	159	7.97E-46
Regulation of cellular metabolic process	187	5.51E-20	127	1.92E-17
Regulation of macromolecule metabolic process	173	5.31E-17	121	1.72E-16

### Canonical pathway associated with PML target genes and TNF*α *responsive genes

We further analyzed significantly altered (*q *< 0.01) canonical pathways defined by PML knockdown and TNF*α *treatment using Kyoto Encyclopedia of Genes and Genomes (KEGG) database (Figure 2d). The top 20 (by gene numbers) are shown in Tables 2 and 3. Consistent with the GO.BP analyses, we found that the largest category of genes (n=95) affected by PML knockdown are those involved in *metabolic pathways* (Table 2). PML is known as a tumor suppressor, and indeed, we identified *Pathways in cancer* as the second largest affected category of genes (n=39) following its knockdown. Similarly, TNF*α *treatment showed that *Metabolic pathways* and *Pathways in cancer* were the top two affected pathways (Table 3). Our pathway analysis also suggested that PML knockdown and TNF*α *treatment commonly affected pathways involved in *cell communication* biological process including *Cytokine-cytokine receptor interaction*, *Focal adhesion*, *Regulation of actin cytoskeleton*, *Endocytosis*, *Tight junction*, *Chemokine signaling pathway*, *Cell adhesion molecules (CAMs)*, *Leukocyte transendothelial migration*, and *Axon guidance* (Tables 2 and 3).

**Table 2:** Top 20 KEGG pathways based on gene number following PML knockdown

**KEGG canonical pathway name**	**Gene number**	**Over-represent fold**	***q *value**
Metabolic pathways	95	3.129177	2.67E-20
Pathways in cancer	39	4.257452	1.77E-12
Cell cycle	30	8.4032	1.78E-17
MAPK signaling pathway	27	3.600042	2.30E-07
Cytokine-cytokine receptor interaction	24	3.297377	4.66E-06
Focal adhesion	23	4.11424	2.30E-07
Regulation of actin cytoskeleton	23	3.847576	7.25E-07
Small cell lung cancer	20	8.4032	7.25E-12
Purine metabolism	20	4.723367	2.30E-07
Endocytosis	20	3.864573	3.69E-06
p53 signaling pathway	19	9.949875	1.71E-12
Pyrimidine metabolism	17	6.33272	3.70E-08
Tight junction	17	4.584133	3.09E-06
Natural killer cell mediated cytotoxicity	17	4.483751	3.65E-06
Chemokine signaling pathway	17	3.199343	0.000168
Cell adhesion molecules (CAMs)	15	4.044824	4.55E-05
Lysosome	14	4.398892	3.96E-05
Leukocyte transendothelial migration	14	4.25103	4.86E-05
Axon guidance	14	3.921493	0.000105
T cell receptor signaling pathway	13	4.349434	7.40E-05

**Table 3:** **Top 20 KEGG pathways based on gene number following TNF*α *treatment**

**KEGG canonical pathway name**	**Gene number**	**Over-represent fold**	***q*Value**
Metabolic pathways	39	2.125783	9.13E-05
Pathways in cancer	35	6.322678	5.29E-16
Cytokine-cytokine receptor interaction	33	7.502728	5.05E-17
Cell adhesion molecules (CAMs)	22	9.817002	4.87E-14
Chemokine signaling pathway	22	6.851449	5.71E-11
Focal adhesion	21	6.216256	8.39E-10
MAPK signaling pathway	20	4.412876	5.87E-07
Tight junction	17	7.585865	2.33E-09
Small cell lung cancer	16	11.12455	4.05E-11
Toll-like receptor signaling pathway	16	9.472391	3.54E-10
Regulation of actin cytoskeleton	15	4.152394	3.88E-05
Axon guidance	14	6.489322	5.87E-07
Leukocyte transendothelial migration	13	6.532169	1.44E-06
Epithelial cell signaling in Helicobacter pylori infection	12	10.55196	2.34E-08
Neurotrophin signaling pathway	12	5.649871	1.69E-05
Hematopoietic cell lineage	11	7.474308	3.16E-06
Natural killer cell mediated cytotoxicity	11	4.801016	0.000148
Endocytosis	11	3.517322	0.001595
ECM-receptor interaction	10	7.118389	1.62E-05
Antigen processing and presentation	10	6.718479	2.42E-05

### Implication of PML and TNF*α *signaling in diseases

**Figure 3:**
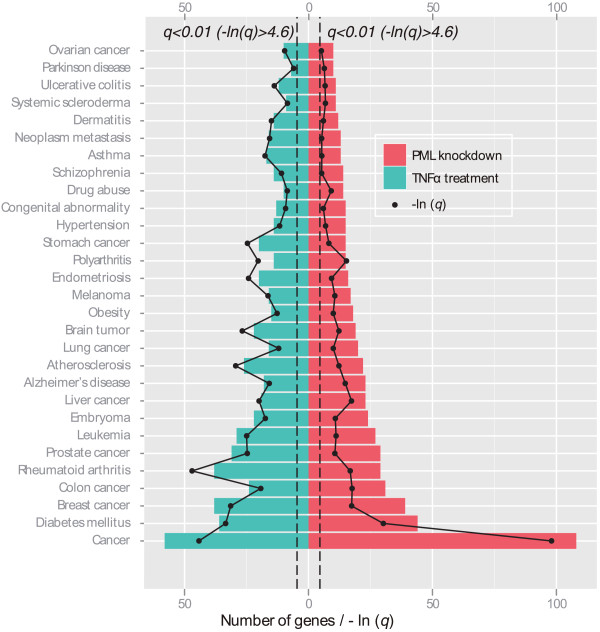
**PML function and TNF*α *signaling are linked to multiple human diseases.** The significantly affected genes by PML knockdown and TNF*α *signaling are significantly associated (*q *< 0.01) with multiple human diseases analyzed as described in Methods using a Disease Ontology database. The diseases are sorted vertically according to the number of associated significantly altered genes following PML knockdown. The transformed values (−*ln*(*qvalue*)) of FDR adjusted *p* value for each category of diseases are shown in the same graph. The dashed lines indicate −*ln*(*q *= 0.01) ≈ 4.6.

PML is a well-defined tumor suppressor; however its role in diseases other than cancers has yet to be determined. In addition, it is well known that endothelium actively participates in cardiovascular and chronic inflammation-related diseases. Because PML is highly expressed in ECs and inflamed tissues, we suspect that PML plays a significant role in regulating the EC physiology in the pathogenesis of EC-related diseases. Therefore, we analyzed the significantly altered genes using Disease Ontology Lite (DOLite) database [16]. We found that PML target genes are significantly associated (*q *< 0.01) with a variety of human diseases (Figure 3). Consistent with its known tumor suppressor role, we identified *Cancer* as the largest category of disease associated with PML. Specifically, we identified cancers of multiple organ origins to be associated with PML expression including breast, colon, prostate, leukemia, embryoma, liver, lung, brain, melanoma, endometriosis, stomach, and ovarian cancers. We also found PML to be linked to cancer metastasis as identified by an association with *Neoplasm metastasis*. Interestingly, PML appears to be involved in several metabolism-related diseases and cardiovascular diseases such as *Diabetes mellitus*, *Obesity*, *Atherosclerosis* and *Hypertension*. PML is also associated with inflammation and auto-immune related diseases including *Rheumatoid arthritis*, *Atherosclerosis*, *Obesity*, *Polyarthritis*, *Asthma*, *Systemic scleroderma*, *Ulcerative colitis*, *Dermatitis*. Our data also show that PML is associated with neural system diseases including *Alzheimer’s diseases*, *Schizophrenia* and *Parkinson’s diseases* and embryonic development disease (*Congenital abnormality*). Consistent with results from GO.BP and KEGG analyses showing that PML and TNF*α *signaling are involved in a similar spectrum of biological processes and canonical pathways (Figure 2, Tables 2 and 3), TNF*α* responsive genes are also significantly associated (*q *< 0.01) with these diseases.

### PML and class I major histological complex human leukocyte antigens

Among the significantly affected genes, we found that a group of class I major histological complex (MHC) human leukocyte antigens (HLAs) were down-regulated by PML knockdown, including HLA-A (5.59 fold), HLA-B (4.71 fold), HLA-C (1.93 fold), HLA-F (1.69 fold), and HLA-G (1.61 fold). Interestingly, this cluster of genes is located on chromosome 6p21.3, a region previously linked to leukemia and other auto-immune diseases [17-22]. Other disease-associated chromosomal regions including 2q36.3–37.1, 6p21.3–22.2, 12q13–14, 12q24.31–33, and 20q11.21–23 also contains PML-knockdown responsive genes and summarized in Table 4. These regions have been previously linked to a variety of human health conditions including multiple cancers, chronic inflammatory diseases, auto-immune diseases, diabetes, cardiovascular disease and developmental defects (Additional file 3: Figure S1). However, the link between PML-regulated gene expression and the disease-associated chromosomal abnormalities is currently unclear.

**Table 4:** Disease-associated chromosomal regions containing PML target genes

**Chromosome region**	**Related health conditions and references**	**Genes in region and fold change**		
		**Symbol**	**siP1-siC**	**siP2-siC**
		ACSL3	0.59	0.44
		DOCK10	0.48	0.53
		C2orf33	0.47	0.58
	2q37 deletion syndrome (intellectual disability, autism, short	SLC16A14	0.63	0.64
2q36.3–37.1	stature, obesity, and characteristic facial features) (Casas et al.	CAB39	0.42	0.58
	2004)	ITM2C	0.23	0.34
		HTR2B	0.65	0.58
		ATG16L1	0.63	0.57
		MAPK13	0.55	0.50
		TAPBP	0.26	0.50
		NOTCH4	0.21	0.22
		CFB	0.59	0.55
		HSPA1B	0.66	0.48
		HLA-B	0.27	0.16
	leukemia, lymphoma, other cancers, multiple sclerosis, chronic	HLA-C	0.59	0.45
	inflammatory arthritis, type I diabetes, psoriasis, other	LTB	0.65	0.65
6p21.3–22.2	autoimmue diseases. (Sawcer *et al* 1996, Bevan *et al* 2000,	HCP5	0.34	0.26
	Concannon *et al* 2005, Maleno *et al* 2005, Qiao *et al* 2007,	HLA-A	0.15	0.21
	Slager *et al* 2011, Conde *et al* 2010)	HLA-G	0.60	0.65
		PRR3	0.66	0.61
		HLA-F	0.64	0.54
		ZNF193	0.56	0.65
		HIST1H4K	0.36	0.23
		HIST1H2BD	0.45	0.44
		TRIM38	0.65	0.61
		TMEM106C	2.06	1.94
		FKBP11	2.44	2.06
		TROAP	2.50	1.55
		KRT7	5.10	3.58
		MFSD5	2.09	1.92
		TARBP2	1.67	1.76
		ATP5G2	2.11	1.53
12q13–14	Intimal sarcoma, lymphoma, gliomas. (Reifenberger *et al* 1994,	BLOC1S1	2.12	1.95
	Rao *et al* 1998, Bode-Lesniewska *et al* 2001)	CDK2	4.87	3.73
		FAM62A	1.65	1.69
		STAT6	1.63	1.58
		SHMT2	1.78	1.88
		CDK4	2.02	2.29
		CTDSP2	2.37	2.21
		FAM119B	2.79	2.82
		MSRB3	3.36	4.34
		RPLP0	1.92	2.05
		MLEC	2.19	1.84
	Teratocarcinoma, dysmorphic features and developmental	DENR	2.14	2.93
	delay, beckwith-wiedemann syndrome, mitochondrial	FAM101A	1.77	2.08
12q24.31–33	myopathy, asthma. (Deyo *et al* 1998 , Casas *et al* 2004,	ZNF664	2.84	3.49
	Brasch-Andersen *et al* 2006, Baple *et al* 2010, Al-Zahrani et al	STX2	3.77	3.51
	2011)	POLE	3.74	2.56
		PXMP2	1.79	1.67
		PLAGL2	1.82	1.70
		TPX2	4.68	3.11
20q11.21–23	Bladder cancer, type II diabetes,myocardial infarction.	C20orf127	2.07	2.10
	(Tanahashi *et al* 2006, Potter *et al* 2008, Sherva *et al* 2008)	BLCAP	2.07	1.67
		TGM2	7.82	7.35
		C20orf129	4.15	2.82

### Crosstalk between PML knockdown and TNF*α *signaling

Given that TNF*α* potently induces PML expression, we anticipated that TNF*α *and PML would regulate expression of a common sets of genes and not just common pathways. Indeed, our microarray gene expression analyses show that this is the case. Intriguingly, we also identified a group of genes (n=166) whose expressions were interactively altered when PML was knocked down in the presence of TNF*α *treatment (Figure 1d). The top 15 such genes and their fold changes are listed in Table 5. Gene ontology information analyses revealed that these genes are involved in KEGG pathways including *Metabolic pathways*, *Cell cycle*, *p53 signaling pathway*, *Hematopoietic cell lineage*, *Apoptosis* and immune response-related KEGG pathways such as *Cytokine-cytokine receptor interaction*, *Graft-versus-host disease* and *Type I diabetes mellitus* (Table 6).

**Table 5:** **Top 15 genes interactively regulated by PML knockdown and TNF*α *treatment**

**Refseq**	**Entrez**	**Symbol**	**Definition**	**siC.T-siC.U**	**siP.T-siP.U**	**siP.U-siC.U**	**siP.T-siC.T**	**siP∗T**
NM_002996.3	6376	CX3CL1	Chemokine (C-X3-C motif) ligand 1	8.88	88.86	0.14	1.40	10.00
NM_006398.2	10537	UBD	Ubiquitin D	7.74	73.62	0.95	9.07	9.51
NM_000450.1	6401	SELE	Selectin E (endothelial adhesion molecule 1)	33.57	293.72	0.78	6.83	8.75
NM_020311.1	57007	CMKOR1	Chemokine orphan receptor 1	1.30	9.84	0.26	1.99	7.56
NM_001007595.1	388125	NLF2	Nuclear localized factor 2	1.40	8.14	0.60	3.48	5.79
NM_152851.1	64231	MS4A6A	Membrane-spanning 4-domains, subfamily A, member 6A, transcript variant 3	0.18	0.99	0.23	1.27	5.51
NM_006623.2	26227	PHGDH	Phosphoglycerate dehydrogenase	0.19	0.98	0.51	2.61	5.17
NM_052941.2	115361	GBP4	Guanylate binding protein 4	2.82	14.26	0.26	1.31	5.05
NM_006332.3	10437	IFI30	Interferon, gamma-inducible protein 30	1.64	8.07	0.60	2.97	4.93
NM_000963.1	5743	PTGS2	Prostaglandin-endoperoxide synthase 2	6.42	29.70	0.32	1.48	4.63
NM_004915.3	9619	ABCG1	ATP-binding cassette, sub-family G, member 1	1.02	3.74	0.34	1.25	3.68
NM_001024465.1	6648	SOD2	Superoxide dismutase 2, mitochondrial, nuclear gene encoding mitochondrial protein	14.96	54.60	0.85	3.08	3.65
NM_003046.2	6542	SLC7A2	Solute carrier family 7 (cationic amino acid transporter, y+ system), member 2	25.69	93.30	0.76	2.75	3.63
NM_002970.1	6303	SAT1	Spermidine/spermine N1-acetyltransferase 1	1.01	3.56	0.62	2.18	3.53
NM_003855.2	8809	IL18R1	Interleukin 18 receptor 1	2.23	7.74	0.63	2.18	3.46
NM_017413.3	8862	APLN	Apelin	1.28	0.18	3.71	0.52	0.14
NM_001255.1	991	CDC20	CDC20 cell division cycle 20 homolog (S. cerevisiae)	0.98	0.16	35.74	5.99	0.16
NM_199160.1	26468	LHX6	LIM homeobox 6	0.49	0.10	0.95	0.19	0.20
NM_001067.2	7153	TOP2A	Topoisomerase II alpha	0.90	0.19	15.98	3.32	0.21
NM_005733.1	10112	KIF20A	Kinesin family member 20A	0.99	0.22	8.03	1.76	0.22
NM_016242.2	51705	EMCN	Endomucin	0.38	0.09	0.59	0.14	0.23
NM_004701.2	9133	CCNB2	Cyclin B2	1.04	0.25	10.86	2.53	0.24
NM_018136.2	259266	ASPM	Asp (abnormal spindle)-like, microcephaly associated (Drosophila)	0.92	0.22	10.06	2.41	0.24
NM_181803.1	11065	UBE2C	Ubiquitin-conjugating enzyme E2C	0.96	0.23	24.06	5.95	0.24
NM_003186.3	6876	TAGLN	Transgelin	3.67	0.91	0.85	0.23	0.27
NM_015234.3	221395	GPR116	G protein-coupled receptor 116	1.04	0.29	1.07	0.30	0.28
NM_014750.3	9787	DLG7	discs, large homolog 7 (Drosophila)	1.00	0.30	6.53	1.96	0.30
NM_003981.2	9055	PRC1	Protein regulator of cytokinesis 1	0.99	0.29	9.94	2.92	0.30
NM_016359.2	51203	NUSAP1	Nucleolar and spindle associated protein 1	0.97	0.29	11.76	3.40	0.30
NM_018131.3	55165	CEP55	centrosomal protein 55kDa	1.00	0.31	9.62	2.91	0.30

**Table 6:** **Top KEGG pathways interactively regulated by PML knockdown and TNF*α *signaling**

**KEGG canonical pathway name**	**Gene number**	**Over-represent fold**	***p *Value**
Metabolic pathways	9	2.29839	0.0944
Cell cycle	7	15.20184	2.92E-05
Cytokine-cytokine receptor interaction	6	6.391213	0.012504
p53 signaling pathway	3	12.18035	0.037604
Hematopoietic cell lineage	3	9.550505	0.037604
Apoptosis	3	9.443196	0.037604
Glycine, serine and threonine metabolism	2	18.67654	0.041913
Prion diseases	2	15.56379	0.053207
Graft-versus-host disease	2	13.34039	0.064162
Type I diabetes mellitus	2	12.73401	0.064162

### Knockdown of PML promotes TNF*α*-induced inflammatory response

**Figure 4:**
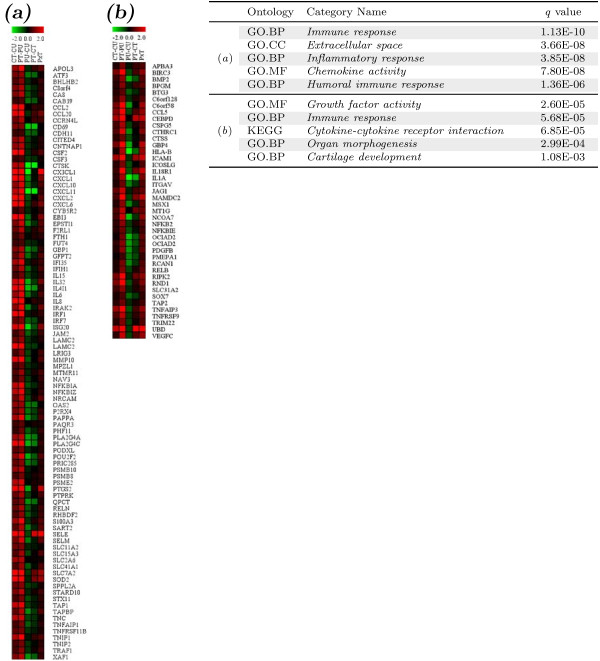
**The interactive effects of PML knockdown on TNF*α *signaling-induced inflammatory response.** (**a** and **b**) Two clusters of genes annotated with inflammation-associated ontology information were identified in a clustering analysis of the log fold change values of significantly affected genes combined with their gene ontology information (GO and KEGG). The affected categories of ontology information and the Fisher exact test results are summarized in the table. “CT-CU”, TNF*α *effects with control siRNA transfection; “PT-PU”, TNF*α *effects with PML siRNAs transfected; “PU-CU”, PML knockdown effects; “PT-CT”, PML knockdown effects when TNF*α *treated; “PxT”, the interaction effects of PML knockdown and TNF*α *treatment defined as *P*×*T *= (*PT*−*PU*)−(*CT*−*CU*)=(*PT*−*CT*)−(*PU*−*CU*). GO, Gene Ontology database; BP, biological function; CC, cellular component; MF, molecular function; KEGG, Kyoto Encyclopedia of Genes and Genomes.

TNF*α* is a cytokine that mediates inflammatory response during wounding, chronic inflammation, and bacterial infection. Our findings suggesting that PML is associated with inflammation and auto-immune related diseases implies that PML may actively participated in TNF*α*-mediated effects in the inflammatory response. To test this, we utilized a cluster analysis combined with the gene functional ontology information (GO and KEGG databases) to examine potential crosstalk between PML and TNF*α*. We identified two clusters of genes significantly associated (*q*≦1.03*E*−3) with inflammation-related ontology information (GO:0006955, GO:0005615, GO:0006954, GO:0008009, GO:0006959, GO:0008083, GO:0009887, GO:0051216 and KEGG pathway *Cytokine-cytokine receptor interaction*). These genes showed a positive synergistic effect between PML knockdown and TNF*α *treatment (Figure 4).

We found that these two clusters of genes are mostly induced by TNF*α* treatment (Figure 4a–b, columns of “CT-CU”). The NF-*κ*B pathway is a well-established early activation pathway downstream of TNF*α *signaling during the inflammatory response. Both of these two clusters contain a large number of genes that are NF-*κ*B activators or target genes. For example, the TNF*α *induced genes, *APOL3*, *TNFAIP1*, *TRAF1* are activators of NF-*κ*B signaling while many other genes are known NF-*κ*B target genes, such as *CCL2*, *CCL5*, *CD69*, *CSF2*, *CSF3*, *ICAM1*, *IL15*, *IL1A*, *IL6*, *IL8*, *IRF1*, *IRF7*, *MMP10*, *NFKB1A*, *NFKB1E*, *NFKB1Z*, *NFKB2*, *PDGFB*, *PTGS2*, *RELB*, *SELE*, *SOD2*, *TAP1*, *TNC*, *TNFAIP1*, *TNFAIP3*, *TNFRSF9*, *TNFRSF11B*. Most interestingly, we found that PML is required for the normal expression of these TNF*α*-induced genes (Figure 4a–b, columns of “PU-CU”) as knockdown of PML decreased expression of these genes. Since members of *TNF* superfamily and *TNF* receptor superfamily play pivotal roles in the activation of NF-*κ*B pathway, we examined the expression of these genes in the microarray and found that PML knockdown did not significantly affect the expression of the *TNF* gene or its superfamily members *TNFSF*7–9, 11–15, and 18, although knockdown of PML did affect expression of *TNFSF4* (2.63*↑*) and *TNFSF10* (2.07*↓*). We also found that knockdown of PML up-regulated a group of TNF receptor superfamily (*TNFRSF*) members, including *TNFRSF1B* (2.21 fold), *TNFRSF10B* (2.49 fold), *TNFRSF10D* (3.42 fold) and *TNFRSF21* (3.62 fold), and down-regulated *TNFRSF10A* (1.75 fold). Other *TNFRSF* members 1A, 4, 6B, 7–9, 10A, 10C, 11A, 11B, 12A, 13B, 13C, 14, 17–19, 19L, and 25 were not significantly affected by PML knockdown.

To determine the effects of PML knockdown in TNF*α *signaling, we compared the TNF*α *effects without and with PML knockdown. We found that the TNF*α*-induced genes were further up-regulated when PML was knocked down (Figure 4a–b, comparing columns between “PT-PU” and “CT-CU”). We also examined the effects of TNF*α* when PML was knocked down and found that TNF*α *treatment relieved or even reversed the suppression of these clusters of genes by PML knockdown (Figure 4a–b, comparing columns of “PT-CT” and “PU-CU”). Therefore, we conclude that these inflammatory response-related genes are interactively regulated by PML and TNF*α *in a positive manner (Figure 4a–b, column of “PxT”) and that PML may have effects on NF-*κ*B activity in TNF*α*-treated HUVECs.

### PML and TNF*α *signaling interactively regulates cell adhesion in ECs

**Figure 5:**
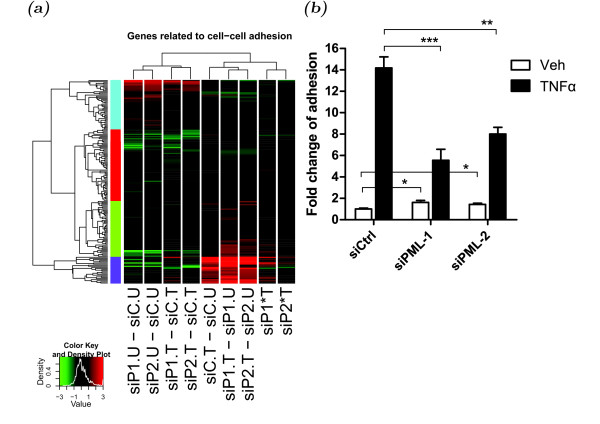
**The effects of PML knockdown and/or TNF*α *on leukocyte:HUVEC adhesion.** (**a**) Clustering analysis of the log change fold values of the coregulatory network of genes significantly affected by PML knockdown and/or TNF*α *signaling. Using *dynamicTreeCut* R package as described in Methods, 4 sub-clusters of significantly altered genes were identified and annotated by color side bars (blue, red, green and purple respectively). Control siRNA, “siC”; two PML siRNAs, “siP1” and “siP2”; vehicle treated samples, “U”; TNF*α *treated samples, “T”; minus sign of “-”, comparison between two samples; sign of “∗”, interaction effects. (**b**) Quantification of the adherence of human leukocyte U937 cells on HUVECs transfected with control siRNA (siCtrl) or two independent PML siRNAs (siPML-1 and siPML-2) without or with TNF*α *treatment. Two-tail unpaired *t*-tests: ∗, *p *< 0.05; ∗∗, *p *< 0.01; ∗∗∗, *p *< 0.001.

Our GO.BP and KEGG analyses suggest that PML knockdown affected *Cell communication* processes and the related KEGG pathways. In-depth analyses of the genes altered by PML knockdown showed that PML knockdown interferes with a molecular network of genes involved in the cell adhesion, cytoskeleton, and signaling transduction by extracellular cytokines/chemokines (Additional file 3: Figure S2). Because TNF*α* is known to activate leukocyte adhesion to endothelial cells during inflammation, we suspect that PML target genes and TNF*α *responsive genes are involved in a coregulatory network of cell adhesion. Indeed, by hierarchial cluster analysis we found that an array of genes (n=182) involved in cell adhesion pathways show interaction patterns following PML knockdown and TNF*α *treatment (Figure 5a). We also identified potential regulatory mechanisms of cell adhesion by PML and TNF*α *signaling through analysis of the sub-clusters of genes in the dendritic tree identified by the hierarchial cluster.

We identified 4 sub-clusters of genes that generally represent 4 putative mechanisms by which PML and TNF*α* regulate HUVEC adhesion pathways. In Figure 5a, the sub-cluster annotated by the blue side bar represents a group of genes that were mostly suppressed by PML (up-regulation by PML knockdown, red in “siP1.U-siC.U” and “siP2.U-siC.U”). However, TNF*α* treatment had a mixed effects (either up- or down-regulation, red or green in “siC.T-siC.U”) on these genes. The interaction effects (“siP1∗T” and “siP2∗T”) mildly showed green, which indicates that TNF*α *treatment had negative effects on PML knockdown-induced gene expression. The sub-cluster annotated by the red bar shows a group of genes whose normal expression required PML (down-regulation by PML knockdown, green in “siP1.U-siC.U” and “siP2.U-siC.U”). TNF*α *treatment suppressed most of these genes (green in “siC.T-siC.U”). For these genes, there was little crosstalk (“siP1∗T” and “siP2∗T” showed mostly black). The sub-cluster annotated by the green bar are genes mostly induced by TNF*α *treatment (red in “siC.T-siC.U”) but not potently affected by PML knockdown (mildly green or black in “siP1.T-siC.U” and “siP2.T-siC.U”), except a small fraction of genes that require PML for their normal expression (down-regulation by PML knockdown). Although this small fraction of genes was not very responsive to TNF*α* treatment alone (slightly red in “siC.T-siC.U”), TNF*α *potently induced the expression of these genes after PML was knocked down (red in “siP1.T-siP1.U” and “siP2.T-siP2.U”). The final sub-cluster annotated by the purple side bar are genes induced by TNF*α* (red in “siC.T-siC.U”) but suppressed by PML knockdown (green in “siP1.U-siC.U” and “siP2.U-siC.U”). Some of these genes showed positive interaction (red in “siP1∗T” and “siP2∗T”) and others showed negative interaction effects (green in “siP1∗T” and “siP2∗T”) by PML knockdown and TNF*α* treatment. Taken together, our data indicate that PML and TNF*α *regulate HUVEC cell adhesion in HUVEC through in an interactive manner by regulating overlapping and distinct groups of genes.

One of the key events during leukocyte transendothelial migration is cell adhesion, which we identified as one of the significantly affected pathways by PML knockdown and TNF*α *treatment (Tables 2 and 3). During the initial stage of leukocyte transendothelial migration, the endothelial cells express cell adhesion molecules to adhere the circulating leukocytes in response to inflammatory cytokines, such as TNF*α*. To determine whether PML regulates cell adhesion in this process, we carried out an *in vitro* cell adhesion assay (Figure 5b). We knocked down PML by two different siRNAs in HUVECs followed by treatment with vehicle or TNF*α* for 4 h. A suspension of fluorescence-labeled human leukocyte U937 cells were added to a monolayer of HUVECs for 30 min. After extensive washing, the adherent cells were quantified by reading the fluorescence signal retained by HUVECs. We found that knockdown of PML modestly increased the U937 adherence to HUVECs in the absence of TNF*α *treatment (1.62 ± 0.19 fold, *p *= 0.02 and 1.43 ± 0.11 fold, *p *= 0.02 for two PML siRNAs, respectively). As expected, TNF*α *potently promoted U937 cell adhesion to HUVECs as shown in control siRNA transfected cells (“siCtrl”, 14.18 ± 1.04 fold, *p *= 1.52×10^−6^). Interestingly, knockdown of PML significantly decreased TNF*α*-mediated induction of cell adhesion (only 5.54 ± 1.03 fold, *p *= 0.0004 and 8.00 ± 0.62 fold, *p *= 0.0015 for two PML siRNAs). Taken together, our data demonstrate that PML and TNF*α *regulates cell adhesion in an interactive manner and that PML is required for maximal TNF*α*-induced leukocyte adhesion to HUVECs.

## Discussion

### The off-target effects of siRNA

SiRNA is a commonly used approach to transiently knock down expression from gene of interests to investigate their function. However, some siRNAs have off-target effects. Indeed, our data (Figure 1a) showed that the overlapping affected genes account for 63% and 68% of the total affected genes by two independent PML siRNAs respectively. Our study suggests that careful evaluation of siRNA by using more than one siRNAs is critical for data analysis.

### The role of PML in class I MHC HLA expression

Our study found that PML is required for normal expression of a cluster of class I MHC HLAs, including HLA-A, HLA-B, HLA-C, HLA-F, and HLA-G as PML knockdown significantly reduced the expression of these genes. Normal expression of class I HLAs is essential for adaptive immune responses, cytotoxicity-mediated cancer cell removal, and precise modulation of inflammatory responses. Loss or down-regulation of class I MHC HLAs plays a causative role in etiology of these conditions [17-22]. Because PML is down-regulated in many cancers [9], we suspect the down-regulation of PML and thereby class I MHC HLAs is one of the mechanisms used by cancers to escape the anti-tumor immune response. Thus, PML likely maintains the expression levels of class I HLAs as part of its tumor suppressor activity. Furthermore, class I HLAs are often down-regulated by virus infection [23], consistent with the notion that PML is an anti-viral protein. Loss of PML may increase the vulnerability to viral infection [24]. Therefore, our analysis suggests that PML is a putative novel regulator of class I HLAs and that PML functions to reduce the susceptibility to cancers and other diseases by controlling expression of downstream target genes, including class I HLAs.

### PML in metabolic control of cell physiology

Tumors have been known to alter metabolism pattern since the description of the Warburg effect [25]. Recent studies have linked tumors with obesity. Obesity is also a known risk factor for diabetes and cardiovascular diseases. PML is known for its role as a tumor suppressor protein; however its role in metabolism remains largely unexplored. Our data suggest that PML is involved in multiple aspects of cellular metabolic pathways involving macromolecules, biopolymer, nitrogen compounds and nucleotides. As we were preparing our manuscript, it was reported that PML negatively regulates adipogenesis [26]. When our manuscript was under review, we learned that two latest papers reporting PML’s role in cancer metabolism [27] and stem cell metabolism [28]. Therefore the role of PML in cellular metabolism is an incomplete understood question worth further in-depth investigation.

### PML inhibits cell adhesion and inflammatory response in endothelial cells: PML’s role in vascular angiogenesis unveiled

Endothelial cell has significant relevance in angiogenesis, a critical process during embryology, cancer development and cardiovascular diseases. Angiogenesis is a series of multicellular morphological modification involving cell adhesion, migration and differentiation [29]. Chronic inflammation usually promotes angiogenesis [29-32]. Our data show that PML regulates multiple pathways involved in cell adhesion (Table 2 and Figure 5), the inflammatory response following TNF*α *treatment (Figure 4), and a molecular network of genes involved in cell mobility (Additional file 3: Figure S2). Together, our data suggest that PML is able to regulate several different aspects of angiogenesis. Indeed, PML has been shown to inhibit hypoxia-mediated neoangiogenesis [33]. We have also recently demonstrated that PML is essential for TNF*α*-mediated inhibition of endothelial cell network formation and migration by regulating downstream target gene expression [15]. We believe that further study on PML and TNF*α *target genes will shed a light on specific molecular mechanisms of angiogenesis in cancer and human cardiovascular diseases.

### The networks that are regulated by both PML knockdown and TNF*α *treatment

TNF*α *signaling has been well-studied but remains to be fully elucidated. Our study suggests that PML and TNF*α *signaling regulates genes involved in a similar spectrum of biological processes, molecular functions, cellular components, canonical pathways, and human diseases (Figure 2, Tables 2 and 3, and Figure 3). These analyses suggest that there exists mutual regulatory networks between PML and TNF*α* signaling. Indeed, we identified a set of NF-*κ*B-dependent genes that are uniquely regulated by PML and TNF*α *signaling (Figure 4). We found that knockdown of PML suppressed the expression of these genes in the absence of TNF*α* treatment but enhanced their expression following TNF*α *treatment. We hypothesize that PML functions as a molecular switch in TNF*α *signaling. As such, we reason that the magnitude of PML accumulation following TNF*α* treatment may have profound effects on TNF*α *activity and that ablation of *PML* gene may lead to dysregulation of inflammatory responses. In fact, our microarray analyses demonstrate that knockdown of PML is associated with a network of genes involved in chronic inflammation-related diseases, such as *Rheumatoid arthritis* and *Atherosclerosis* (Figure 3). In summary, we have identified the genes induced by TNF*α *treatment alone, the genes induced only when PML is knocked down and the genes co-regulated by TNF*α *and PML knockdown. We believe our results provide future directions in the study of PML- and TNF*α*-coregulated inflammatory responses.

## Conclusions

Through microarray analyses, we identified novel PML target genes and TNF*α *responsive genes in HUVEC. Intriguingly, we found that PML is required for normal expression of class I MHC HLAs, thereby suggesting a potential novel mechanism by which PML functions as a tumor suppressor. Our gene ontology information analyses show that PML target genes and TNF*α *responsive genes participated in a variety of overlapped and distinct biological processes, canonical pathways and human diseases. Notably, we identified clusters of genes implicated in inflammation-related diseases and TNF*α*-elicited NF-*κ*B-mediated immune responses that are synergistically regulated by PML knockdown and TNF*α *treatment. Our data further suggests that PML is a putative novel TNF*α* regulator, required to finely control TNF*α*-mediated inflammatory responses. Our study adds to the understanding of TNF*α *biology and provides novel information for potential pharmaceutical targets in TNF*α*-related diseases. Through clustering analyses, we noticed that the adhesion-related crosstalk between PML and TNF*α *engages a complex molecular mechanism. Indeed, our leukocyte adhesion assays demonstrated that the effects of PML knockdown could be switched from activation to inhibition of leukocyte adhesion depending on the presence or absence of TNF*α*. Together, our results that had not been previously appreciated have uncovered roles of PML and its crosstalk with TNF*α *signaling.

## Methods

### Experimental design

A 3×2 factorial design was adapted. To study PML gene function, we used an siRNA-mediated knockdown approach. The siRNA factor had three levels: control siRNA (siC, no knockdown) and two different PML siRNAs (siP1 and siP2). The two PML siRNAs were used to eliminate off-target effects. TNF*α *treatment had two levels: no treatment (U) and treatment (T). Each sample had technical duplicates on different microarray chips.

### Sample preparation and the microarray

To prepare the samples, HUVECs (Lonza, C2519A, passage *# *< 5) were transfected with a control siRNA (Dharmacon, D-001810-01) or two different PML siRNAs (Dharmacon, J-006547-05 and J-006547-07) for 72 h followed by treatment with vehicle (water) or 20 *ng*/*mL*TNF*α* for 20 h. Total RNA was extracted with a USB PrepEase kit following the manufacturer’s instructions. An aliquot of the total RNA was reverse transcribed into cDNAs and verified by qRT-PCR and gel electrophoresis to ensure that the knockdown efficiency of PML was greater than 75%. The mRNAs were reverse-transcribed into biotin labeled cRNAs with the MessageAMP II kit (Ambion) prior to the microarray hybridization with Human Reference Sequence-8 Version 2 Expression BeadChip (Illumina, HumanRef-8_V2_0_R0_11223162_A). The probe-level raw data with background noise subtracted were used for statistical analyses. The quality controls of our microarray are shown in supplementary data (Additional file 3: Figure S3). The microarray was processed by Genomics Core Facility at Cleveland Clinic Foundation. The background-subtracted raw data is enclosed as Additional file 4.

### Data preprocessing, gene lists and functional analyses

The microarray data were analyzed in the R/Bioconductor environment [34,35]. Briefly, the raw data were preprocessed with the *lumi* package [36,37]. The probe quality was assessed by detection *p*-values. Those probes with non-significant detection *p*-values (*p *> 0.1) in all samples were removed prior to analyses. The data was transformed by a *Variance-Stabilizing Transformation* (*VST*) package [38] and normalized by a *Robust Spline Normalization* (*RSN*) package [38]. Using the *Linear Models for Microarray Data* (*LIMMA*) package [39] and empirical Bayes method with the false discovery rate (*FDR*) adjusted by the Benjamini and Hochberg’s method, we retrieved the significantly changed gene lists (>1.5*fold* and FDR adjusted *p*(*q*) < 0.05). The samples treated by two different PML siRNAs were considered as independent biological samples to retrieve gene lists. The PML knockdown affected gene list was generated by averaging the commonly affected genes by both PML siRNAs. Hierarchical clustering with average linkage was used to for the cluster analysis. Sub-clusters were identified by the *dynamicTreeCut* R package [40]. For functional ontology analyses, we used the hypergeometric test through the *GeneAnswers* package [41]. The chromosomal pattern of altered genes were analyzed by *MicroArray Chromosome Analysis Tool* (*MACAT*) package [42]. The clustering analyses with ontology information was done by *TEASE* (Tree-Ease) in the *MultiExperiment Viewer* (MeV v4.8, an R-based software). The profile of significantly affected genes is shown as a heatmap in supplementary data (Additional file 3: Figure S4).

### Cell adhesion assays

Cell adhesion assays were carried out with an endothelial cell adhesion assay kit (Millipore, ECM645) according to the manufacturer’s instructions. Briefly, HUVECs were transfected with a control non-targeting siRNA or two different PML siRNAs and equally seeded in a 96-well plate until a monolayer of confluent cells was reached. The resulting monolayer of HUVECs were treated with 20 *ng*/*mL*TNF*α* for 4 h, prior to 30-min incubation with U937 human leukocyte cells pre-labeled with Calcein AM^®;^. The plate was washed extensively and the adherent U937 cells were quantified by reading fluorescence of 485*nm*/530*nm* on a microplate reader.

## Abbreviations

PML: Promyelocytic leukemia protein; EC: Endothelial cell; TNFα: Tumor necrosis factor alpha; HUVEC: Human umbilical vein endothelial cell; siRNA: Small interference RNA.

## Competing interests

The authors declare no competing interests.

## Author’s contributions

XC and H-YK designed the experiments. XC performed the experiments and carried out the microarray statistical analyses. XC and H-YK discussed the data and wrote the manuscript. Both authors read and approved the final manuscript.

## Supplementary Material

Additional file 1 **Gene lists.** An excel spreadsheet containing the significantly affected gene lists and the ontology annotation information. Click here for file

Additional file 2 **Affected categories of gene ontology.** An excel spreadsheet containing the significantly affected gene ontology categories. Click here for file

Additional file 3 **Supplemental data.** A PDF file contains: supplemental materials and methods; **Figure S1,** the chromosomal analysis of PML target genes; **Figure S2,** the molecular network of PML target genes related to cell mobility and cytokine/chemokine signaling. **Figure S3,** the quality controls of microarray samples **Figure S4,** the heatmap and subclusters of the significantly affected genes by PML knockdown and TNF*α *treatment; Click here for file

Additional file 4 **Raw data.** An excel spreadsheet containing the background-subtracted probe level raw data. Click here for file
