# Supplementary material for: Microarray analysis revealing common and distinct functions of promyelocytic leukemia protein (PML) and tumor necrosis factor alpha (TNFα) signaling in endothelial cells
Source: BMC Genomics. 2012 Sep 4;13:453. doi: 10.1186/1471-2164-13-453 (PMC3542097; doi:10.1186/1471-2164-13-453)
Supplement: Additional file 3 — Supplemental data. A PDF file contains: supplemental materials and methods; Figure S1, the chromosomal analysis of PML target genes; Figure S2, the molecular network of PML target genes related to cell mobility and cytokine/chemokine signaling. Figure S3, the quality controls of microarray samples Figure S4, the heatmap and subclusters of the significantly affected genes by PML knockdown and TNFα treatment; [file 1471-2164-13-453-S3.pdf]

Additional file 3: Supplemental Data

Xiwen Cheng and Hung-Ying Kao

Materials and Methods

Cell culture, TNFα treatment and siRNA transfection

Human umbilical vein endothelial cells, HUVECs were from Lonza (#C2519A) and cultured in Endothelial cell Growth Medium-2 (EGM-2, Lonza, CC-4176). Lower than 5-passage of cells were used in this study. TNFα was treated with 20 ng/mL for 20 h. The siRNAs were purchased from Dharmacon with non targeting control (D-001810-01), PML (J-006547-05 and J-006547-07). The transfection reagent was DharmaFECT1 (Dharmacon, T-2001).

Microarray data deposit

The microarray data has been deposited to GEO under the series *GSE35000*.

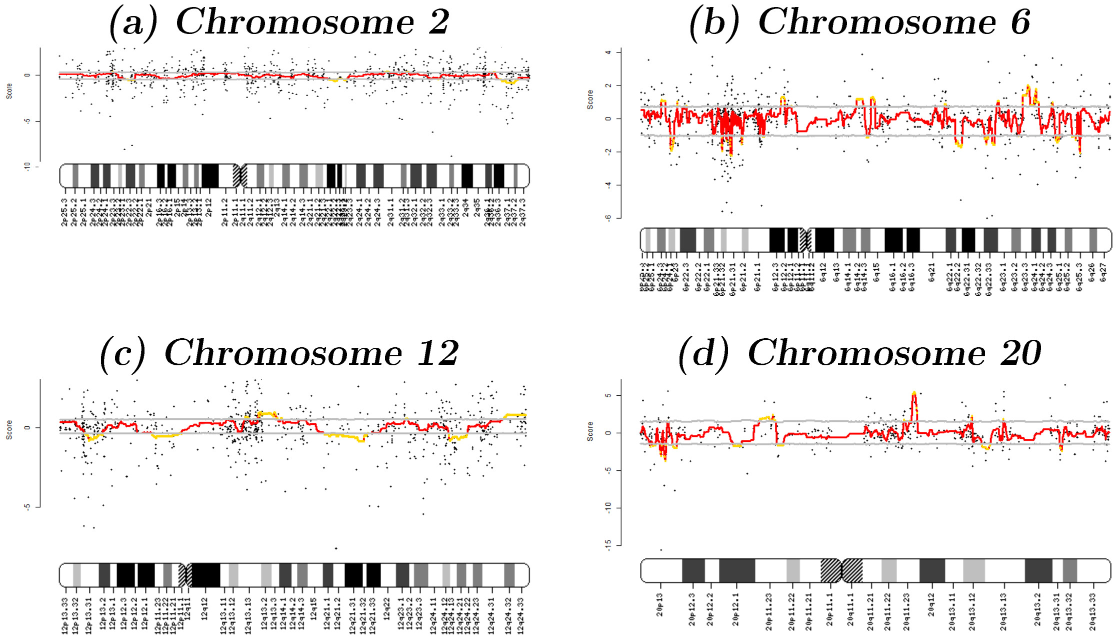

FigS 1. Chromosomal analysis of gene expression following PML knockdown. The gene expression patterns on indicated chromosomes were analyzed by the MicroArray Chromosome Analysis Tool (MACAT) R/Bioconductor package (1). The yellow dots highlight the significantly affected regions of chromosomes with over- or down-expression of genes. Black dots, t-test score for genes; grey lines, the sliding average scores of 0.025 and 0.0975 quartiles; red line, the sliding average permuted score; yellow highlight, regions with scores exceeding the grey lines. By examining the significantly altered gene lists, our data suggest that chromosomes 2q36.3–37.1, 6p21.3–22.2, 12q13–14, 12q24.31–33, and 20q11.21–23 contains clusters of genes showing significantly up- or down-regulation patterns. These regions are also linked to different human diseases. The genes and related diseases are summarized in Tab. 3.

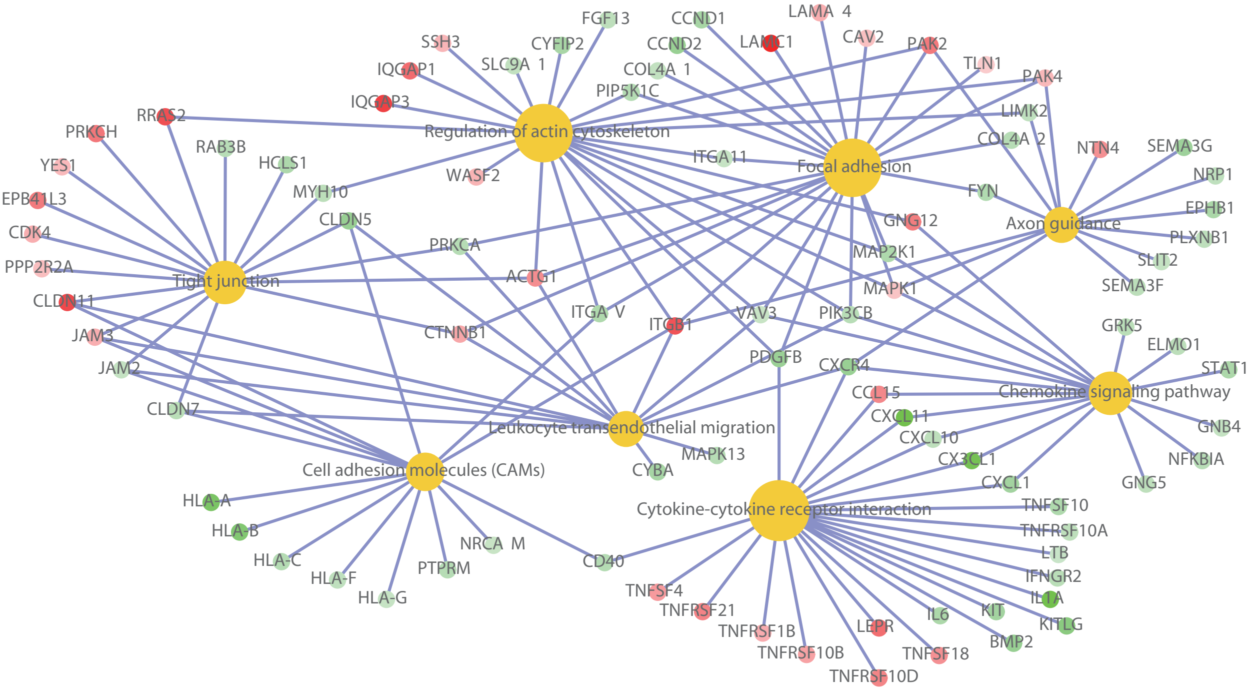

FigS 2. A molecular network of significantly altered genes related to cell mobility and cytokine/chemokine signaling following PML knockdown. Several KEGG pathways are shown including: Tight junction, Cell adhesion molecules (CAMs), Focal adhesion, Leukocyte transendothelial migration, Regulation of actin cytoskeleton, Axon guidance, Chemokine signaling pathway, and Cytokine-cytokine receptor interaction were affected when PML was knocked down. The corresponding significantly altered genes are plotted as dots in color according to the log fold change of expression levels (red, up-regulation; green, down-regulation). The centroid of a pathway is represented as a yellow circle. Its size is correlated with the number of affected genes in that pathway. The graph was generated with “GeneAnswers” R package (2).

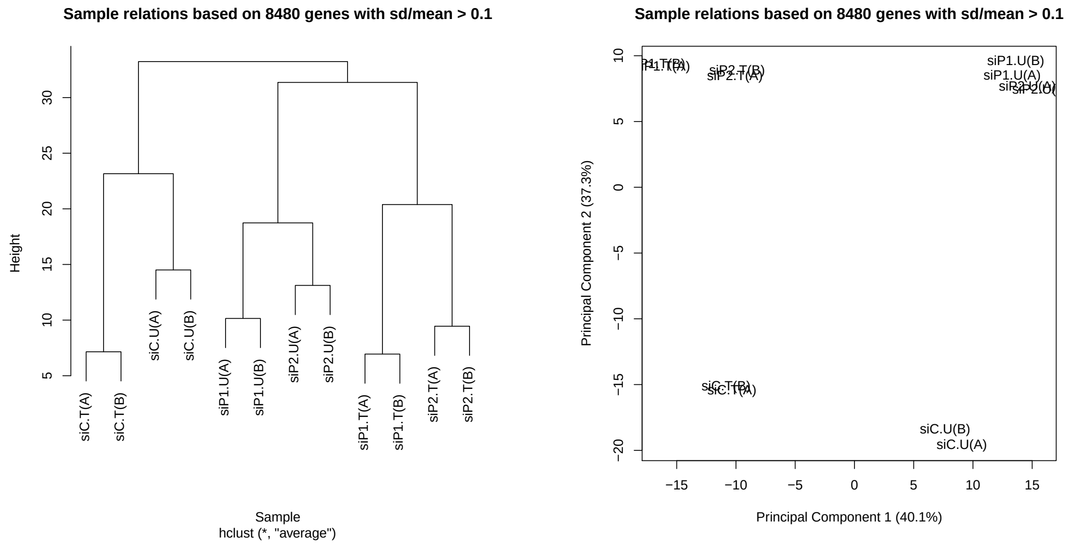

(a) *(b)*

FigS 3. Quality control of microarray samples. (*a*) Hierarchical cluster of the 12 microarray samples after preprocessing. The height represents an arbitrative distance of similarity between two clusters in the dendrogram. (*b*) Multiple dimensional scaling (MDS) analysis with two principal components of the 12 preprocessed microarray samples. The distance between samples is correlated with their similarity. Two different PML siRNAs (siP1, siP2); control siRNA (siC); untreated samples, "U"; TNFα treated samples, "T"; the bracketed letters "(A)" or "(B)", technical duplicates on two different microarray chips. These quality control images were generated using "lumi" package in R/bioconductor (3–7).

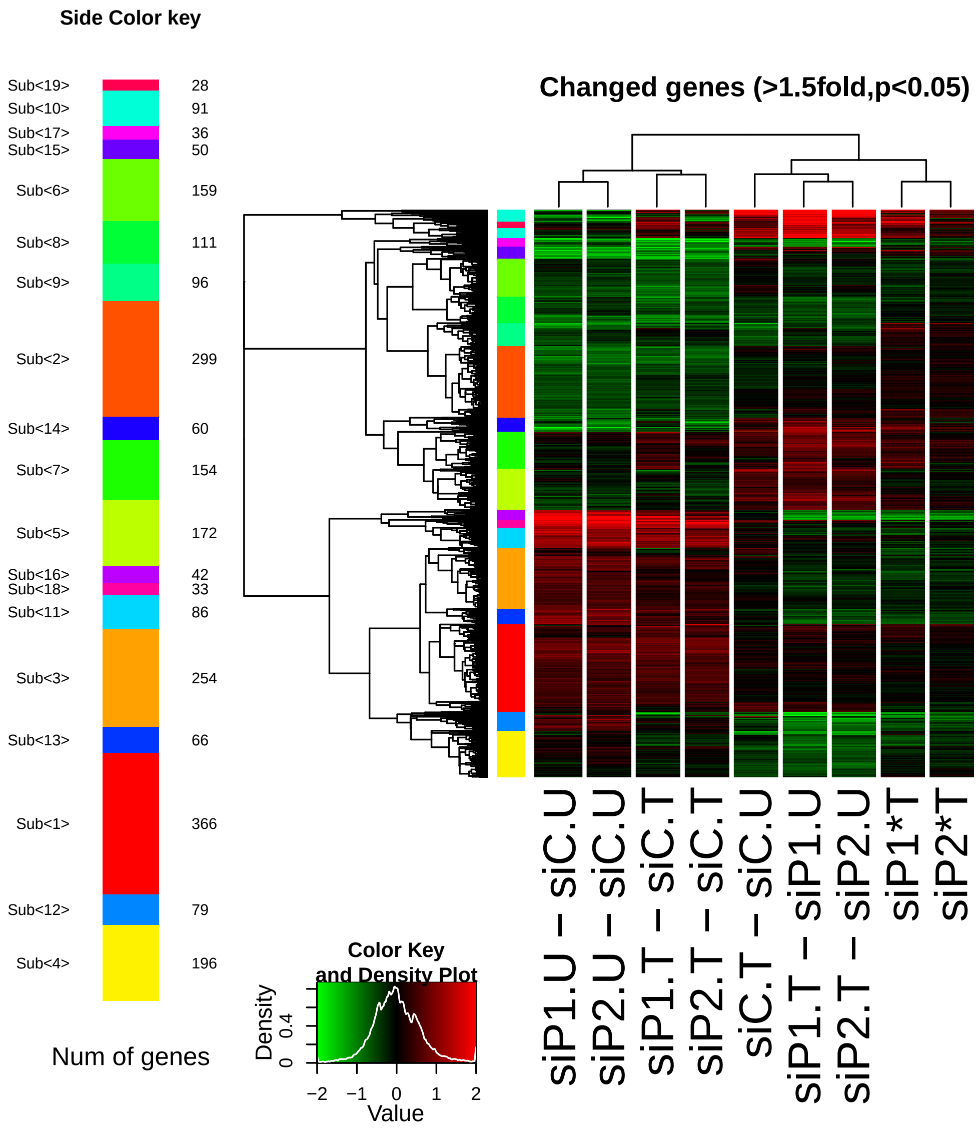

FigS 4. Cluster analysis of the significantly altered genes following PML knockdown and/or TNFα treatment. Log fold change data of the significantly altered genes (>1.5 fold, $p<0.05$) by either PML knockdown or TNFα treatment were subjected to cluster analysis. Both of the columns and rows were clustered with a hierarchical method on average links. The sample labels are described in FigS. 3. The side color key indicates 19 different sub-clusters (Sub<1>–Sub<19>, each with > 20 genes) identified by dynamicTreeCut R package (8) as described in Methods. The gene numbers in each sub-cluster are indicated beside the side color key.

References

[1] Toedling J, Schmeier S, Heinig M, Georgi B, Roepcke S: **MACAT–microarray chromosome analysis tool.** *Bioinformatics* 2005, **21**(9):2112–3.

[2] Feng G, Du P, Krett N, Tessel M, Rosen S, Kibbe W, Lin S: **A collection of bioconductor methods to visualize gene-list annotations**. *BMC Res Notes* 2010, **3**:10.

[3] Du P, Kibbe WA, Lin SM: **nuID: a universal naming scheme of oligonucleotides for illumina, affymetrix, and other microarrays.** *Biol Direct* 2007, **2**:16.

[4] Du P, Kibbe WA, Lin SM: **lumi: a pipeline for processing Illumina microarray.** *Bioinformatics* 2008, **24**(13):1547–8.

[5] Gentleman RC, Carey VJ, Bates DM, Bolstad B, Dettling M, Dudoit S, Ellis B, Gautier L, Ge Y, Gentry J, Hornik K, Hothorn T, Huber W, Iacus S, Irizarry R, Leisch F, Li C, Maechler M, Rossini AJ, Sawitzki G, Smith C, Smyth G, Tierney L, Yang JY, Zhang J: **Bioconductor: open software development for computational biology and bioinformatics.** *Genome Biol* 2004, **5**(10):R80.

[6] Team RDC: *R: A Language and Environment for Statistical Computing.* Vienna, Austria: R Foundation for Statistical Computing 2009.

[7] Lin SM, P D, Huber W, Kibbe WA: **Model-based variance-stabilizing transformation for Illumina microarray data.** *Nucleic Acids Res* 2008, **36**(2):e11.

[8] Langfelder P, Zhang B, Horvath S: **Defining clusters from a hierarchical cluster tree: the Dynamic Tree Cut package for R.** *Bioinformatics* 2008, **24**(5):719–20.
